# Primary lipoblastic nerve sheath tumor in an inguinal lymph node mimicking metastatic tumor: a case report and literature review

**DOI:** 10.3389/fonc.2023.1258769

**Published:** 2023-10-30

**Authors:** Chengxin Chen, Jiachen Cao, Lingxie Song, Wenjie Wang, Dandan Guo, Qi Shi, Ying Zhang, Yunzhao Chen, Chunxia Liu, Feng Li

**Affiliations:** ^1^ Department of Pathology and Key Laboratory for Xinjiang Endemic & Ethnic Diseases, The First Affiliated Hospital, Shihezi University School of Medicine, Shihezi, China; ^2^ Department of Pathology and Medical Research Center, Beijing Institute of Respiratory Medicine and Beijing Chao-Yang Hospital, Capital Medical University, Beijing, China; ^3^ The People’s Hospital of Suzhou National Hi-Tech District, Suzhou, China; ^4^ Department of Pathology, The Second Affiliated Hospital of Guangzhou Medical University, Guangzhou, China

**Keywords:** lipoblastic nerve sheath tumor, lipoblast-like cells, nuclear atypia, lymph node, case report

## Abstract

Lipoblastic nerve sheath tumors of soft tissue are characterized as schwannoma tumors that exhibit adipose tissue and lipoblast-like cells with signet-ring morphology. They have been documented to arise in various anatomic locations, including the thigh, groin, shoulder, and retroperitoneum. However, to our knowledge, this tumor has not been previously reported as a lymph node primary. We present herein the first case of a benign primary lipoblastic nerve sheath tumor arising in an inguinal lymph node in a 69-year-old man. Microscopic examination revealed a multinodular tumor comprising fascicles of spindle cells, as well as adipocytic and lipoblast-like signet-ring cell component in the context of schwannoma. Despite the presence of some bizarre cells with nuclear atypia, no obvious mitotic activity or necrosis was observed. Immunohistochemical analysis showed strong and diffuse expression of S-100, SOX10, CD56, and NSE in the spindle cells as well as in the signet-ring lipoblast-like cells and the mature adipocytes. Sequencing analysis of the neoplasm identified six non-synonymous single nucleotide variant genes, specifically *NF1*, *BRAF*, *ECE1*, *AMPD3*, *CRYAB*, and *NPHS1*, as well as four nonsense mutation genes including *MRE11A*, *CEP290*, *OTOA*, and *ALOXE3*. The patient remained alive and well with no evidence of recurrence over a period of ten-year follow-up.

## 1 Introduction

Benign lipoblastic nerve sheath tumors are an extremely rare subset of neural neoplasms, initially described by Plaza in 2006 (1). They are distinguished by the coexistence of adipocytic and lipoblast-like signet-ring cell component, in the setting of a schwannoma background. Tumor cells displaying signet-ring or lipoblast-like morphology, however, are mostly associated with malignant epithelial neoplasms, mesotheliomas and melanomas (2–5) and have been only rarely observed in soft tissue spindle cell tumors. Lipoblastic nerve sheath tumors have been identified in several anatomical sites, including the thigh, groin, shoulder, and retroperitoneum, yet there have been no reports of this tumor arising in lymph nodes. Nerve sheath tumors within lymph nodes often raise concern for metastatic malignancies. Here, we present an exceptional instance of lipoblastic nerve sheath tumor that originated from the inguinal lymph node in a 69-year-old man. The clinicopathologic, light microscopic, immunohistochemical, and molecular features of the tumor is described and their differential diagnosis is discussed for raising awareness of this rare and intriguing condition.

## 2 Materials and methods

### 2.1 Immunohistochemistry

The surgical specimen was fixed in 10% buffered formalin, routinely processed and embedded in paraffin, and 4 µm-thick sections were cut for immunohistochemical procedures. The following commercially available primary antibodies were used: S-100(1:1600), SOX10(1:200), Melan-A(1:100), HMB45(1:100), CD56(1:500), NSE (1:1000), CD31(1:200), CD34(1:400), CDK4(1:400), MDM2(1:50), AE1/AE3(1:200), Desmin(1:300), SMA(1:400), Vimentin(1:100), H3K27me3(1:200), INI1 (1:100) and Ki-67 (1:200). These antibodies were obtained from ZSGB-BIO, Beijing, China.

### 2.2 Fluorescence *in situ* hybridization and whole-exome sequencing and mutation calling

Interphase fluorescence *in situ* hybridization (FISH) was performed on formalin-fixed paraffin-embedded (FFPE) sections using a MDM2 dual color break-apart probe at 12q15 and CDK4 dual color break-apart probe at 12q14 (Linked-Biotech Pathology, Guangzhou, China), respectively. The experimental procedures were performed following manufacturer’s instructions. Subsequently, one hundred nonoverlapping tumor cell nuclei were conducted, wherein the average count of MDM2 or CDK4 signals, as well as CEP12 signals, was determined. An MDM2/CEP12 or CDK4/CEP12 ratio ≥2.0 was deemed indicative of gene amplification, signifying an amplified state. Conversely, an MDM2/CEP12 or CDK4/CEP12 ratio <2.0 was indicative of nonamplification.

Genomic DNA was extracted from FFPE tissues using the GeneRead DNA FFPE Kit. the Agilent SureSelect XT Human All Exon V6 Kit (Agilent, Beijing, China) was used for constructing the libraries, followed by next-generation sequencing. During the library construction, the genomic DNA was fragmented, end-repaired, adenylated at the 3’ ends, end-connected, amplified, purified, and size-selected. The library was sequenced on an Illumina X10 platform (Illumina Inc., San Diego, CA, USA). The WES data were analyzed for mutations, using the human genome build hg19 as the reference genome. The SNVs and indels were analyzed using GATK MuTect2. To improve the accuracy of the SNVs, the sequenced reads were realigned to hg19 using the Burrows-Wheeler Aligner (BWA-MEM, http://biobwa.sourceforge.net/). Gene annotation was performed using the Kyoto Encyclopedia of Genes and Genomes (KEGG) database (http://www.genome.jp/kegg/). KEGG pathway enrichment was carried out using KOBAS (version 3.0, default parameters) based on the annotation results.

## 3 Case description

### 3.1 Clinical features

A 69-year-old man with no significant medical history visited the hospital surgical service complaining of a painless mass in his right groin that had been present for four months. The patient reported that the mass had slowly increased in size over the previous weeks. There was no other notable lymphadenopathy or clinical signs of neurofibromatosis. The patient underwent an excisional biopsy of the mass and did not receive any postoperative treatment. Over the course of ten years of follow-up, the patient remained alive and well with no evidence of recurrence (**Supplementary Figure 4**).

### 3.2 Gross features

Macroscopically, the mass was nodular shaped, greyish-red and dark red on the surface, 5.4×2.2×1.9 cm in size with a little adipose tissue, and lobulated cut surface. There was no hemorrhage, necrosis, or cystic degeneration.

### 3.3 Histologic findings

**Figure 1:**
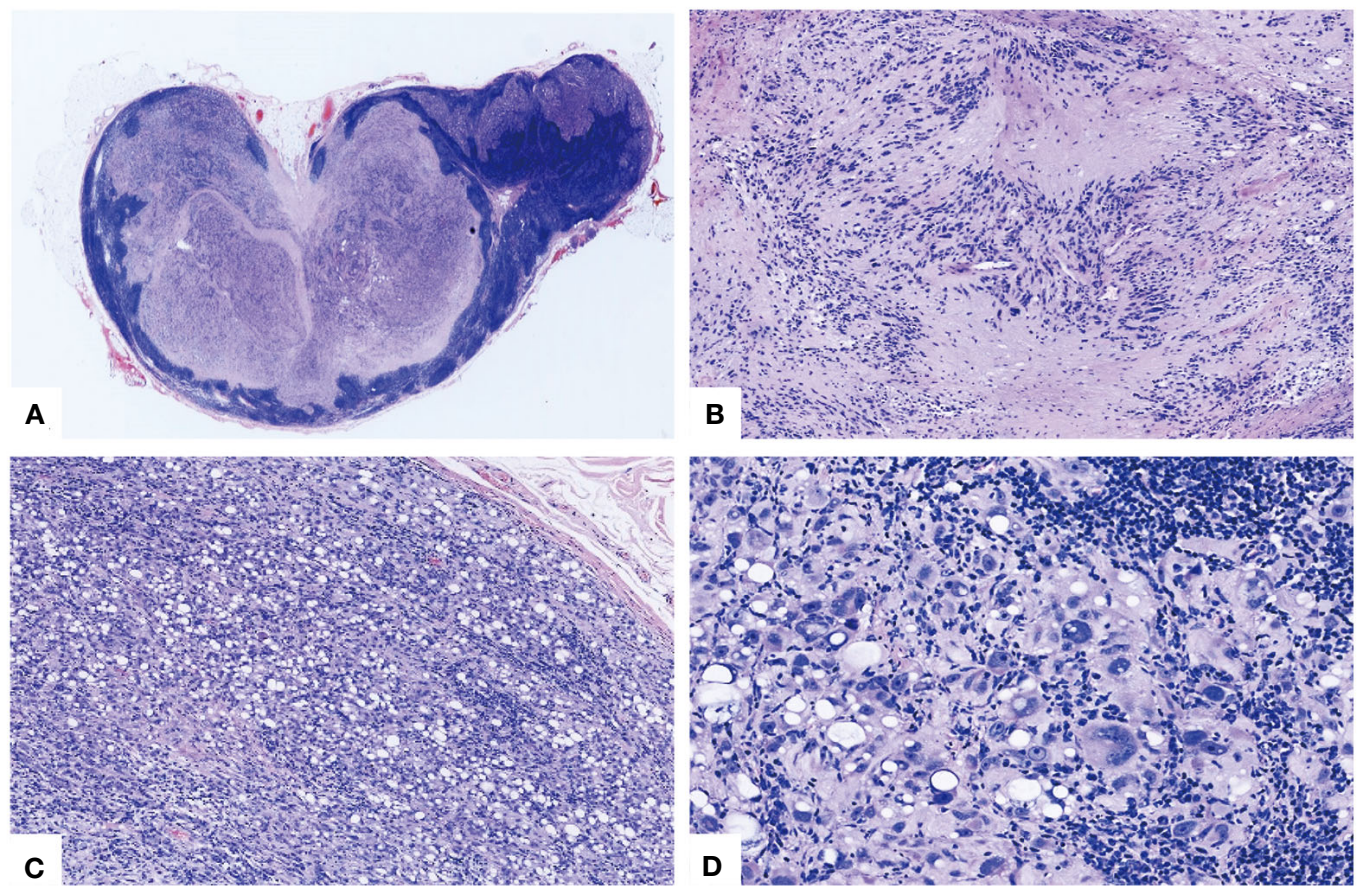
Morphological characteristics of the lipoblastic nerve sheath tumor. **(A)** The vestigial peripheral lymph node tissue underwent compression from a multinodular tumor (HE×20). **(B)** Spindle tumor cells were aligned in intersecting fascicles with nuclear palisades forming Verocay bodies (HE×100). **(C)** The distribution of mature adipocytes and lipoblast-like cells, interspersed with sporadic atypical cells in a disordered pattern (HE×100). **(D)** Certain cells exhibited marked atypia with aberrant nuclei and striking nucleoli, notwithstanding the absence of mitotic activity or necrosis (HE×400).

Microscopic examination revealed the presence of residual peripheral lymph node tissue, which was compressed by a multinodular neoplasm (**Figure 1A**). The neoplasm was composed of moderately cellular spindle cells, arranged in crossing bundles with focal nuclear palisades and embedded within a variably collagenous matrix, forming verocay bodies (**Figure 1B**). Upon further examination, mature adipocytes as well as lipoblast-like cells displaying a signet-ring cell appearance were scattered or densely distributed in certain areas (**Figure 1C**). At higher magnification, the spindle cells exhibited elongated, wavy nuclei with abundant cytoplasm and indistinct boundaries. Some of tumor cells showed obvious atypia with vesicular cytoplasm and bizarre nucleus with prominent nucleoli (**Figure 1D**), but no evidence of mitotic activity or necrosis was observed. Additionally, focal chronic inflammatory infiltrate cells were present. There were no psammoma bodies or melanin pigment deposition identified in the neoplasm.

### 3.4 Immunohistochemical findings

**Figure 2:**
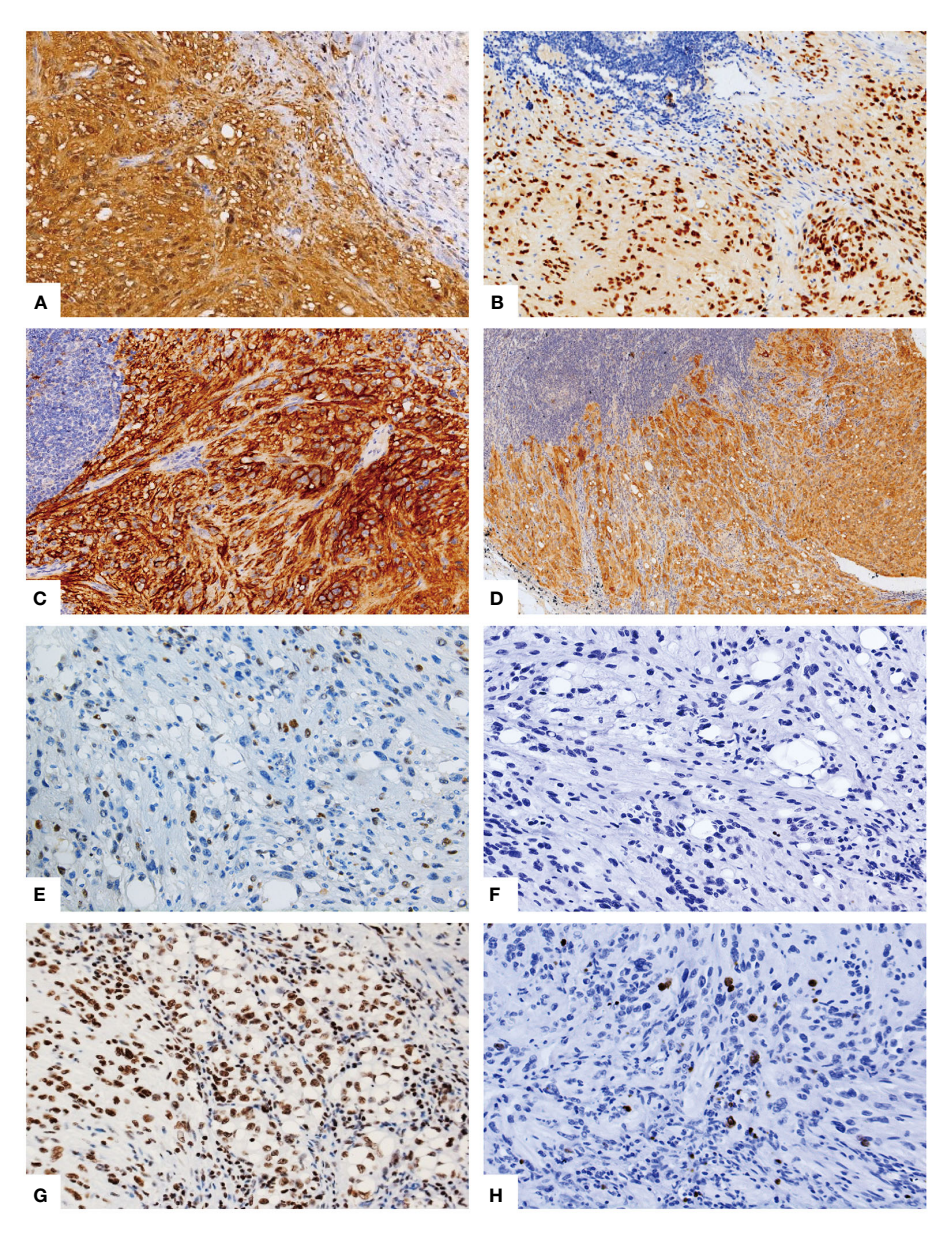
Immunohistochemical phenotype of the lipoblastic nerve sheath tumor. The immunohistochemical analysis demonstrated a diffuse and strong positive staining for S-100 **(A)**, SOX10 **(B)**, and CD56 **(C)** in the spindle cells as well as in the signet-ring lipoblast-like cells and the mature adipocytes. Additionally, Melan-A **(D)** exhibited diffuse positivity. Scattered positive staining for CDK4 **(E)** was observed, while MDM2 **(F)** showed negative staining. Furthermore, the histone 3 trimethyl K27 (H3K27me3) stain revealed nuclear positivity in the tumor cells **(G)**. The proliferative index, as indicated by the Ki-67 staining, was less than 5% **(H)**.

The immunohistochemical analysis conducted on the tumor cells revealed a diffuse expression of S-100 (**Figure 2A**), SOX10 (**Figure 2B**), CD56 (**Figure 2C**), NSE, MelanA (**Figure 2D**), INI1, and vimentin in the spindle cells as well as in the signet-ring lipoblast-like cells. There was focal positivity for CD34. Moreover, the tumor cells were negative for HMB45, AE1/3, EMA, Desmin, SMA and CD31. Notably, the immunostaining for CDK4 (**Figure 2E**) was positive in the scattered tumor cell nuclear, while MDM2 was totally negative (**Figure 2F**). Furthermore, a subsequent investigation utilizing a histone 3 trimethyl K27 (H3K27me3) stain showed diffuse nuclear positivity in the tumor cells (**Figure 2G**). The proliferative index, as determined by Ki-67 was less than 5% (**Figure 2H**).

### 3.5 Molecular findings

**Figure 3:**
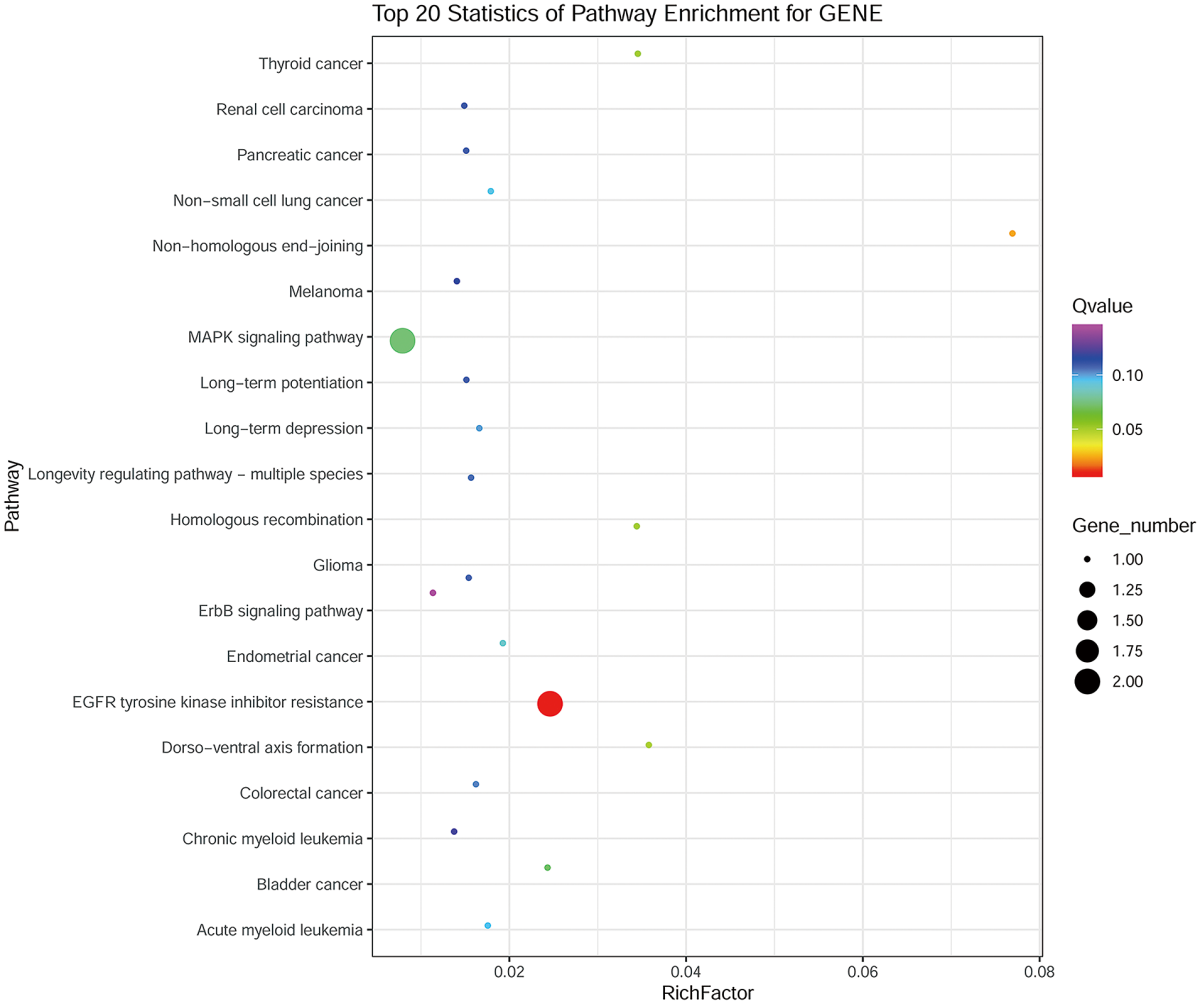
KEGG analysis of the 10 genes identified in the lipoblastic nerve sheath tumor occurring in an inguinal lymph node.

Whole-exome sequencing was performed on 1 case of lipoblastic nerve sheath tumor, 1 of schwannoma, and 1 of malignant peripheral nerve sheath tumor (MPNST) samples, respectively. The results of the sequencing analysis revealed the presence of five nonsynonymous single nucleotide variant (SNV) genes, namely *CFTR, RAG2, SOX3, NF2*, and *F8*, as well as two nonsense mutation genes, namely *MMACHC* and *LDLR*, in the schwannoma sample. Similarly, the MPNST sample was found to harbor one nonsynonymous SNV gene, namely *PRODH*, and two nonsense mutation genes, namely *NF1* and *OTOA*. In the case of the lipoblastic nerve sheath tumor, a total of six nonsynonymous SNV genes (*NF1, BRAF, ECE1, AMPD3, CRYAB*, and *NPHS1*) and four nonsense mutation genes (*MRE11A, CEP290, OTOA*, and *ALOXE3*) were identified. Interestingly, KEGG pathway analysis of these 10 genes demonstrated significant enrichment in two main pathways: EGFR tyrosine kinase inhibitor resistance and MAPK signaling (**Figure 3**).

The FISH analysis revealed the absence of *MDM2* and *CDK4* amplification in this lipoblastic nerve sheath tumor. The MDM2/CEP12 signal ratio was determined to be 1.04, and the CDK4/CEP12 signal ratio was 1.05, as illustrated in **Supplementary Figure 1**.

## 4 Discussion

Benign nerve sheath tumors, also known as schwannomas, are composed entirely or nearly entirely of differentiated neoplastic Schwann cells. These tumors can originate from any myelinated nerve, including the cranial sensory nerves, spinal cord roots, cervical nerves, vagus nerves, peroneal nerves, and ulnar nerves. Although schwannomas can arise from any site, peripheral nerves in the skin and subcutaneous tissues of the head and neck, flexor surfaces of the extremities, and spinal intradural extramedullary examples are the most commonly affected. Lymphatic involvement is a rare and poorly characterized occurrence in schwannomas. To our knowledge, only approximately 20-30 cases of nerve sheath tumors in lymph nodes have been reported in the scientific literature (6–11).

Tumors displaying signet-ring cell morphology typically prompt a well-defined pathologic differential diagnosis. Signet-ring cells are commonly linked to epithelial malignancies, specifically poorly differentiated adenocarcinoma of the stomach or colon (2), prostate cancers (3), lobular carcinoma of the breast (12) and urinary bladder (13). The presence of signet-ring cells in the context of mesenchymal neoplasms is exceedingly rare, with the exception of liposarcoma and melanoma. However, certain vascular tumors, such as epithelioid angiosarcoma and epithelioid hemangioendothelioma, as well as malignant peripheral nerve sheath tumors, may exhibit this morphology (14, 15).

Typically, benign nerve sheath tumors are spindle cell tumors that are encapsulated and composed primarily of well-differentiated Schwann cells, with the majority of cases being biphasic tumors that comprise compact areas (Antoni A) with sporadic nuclear palisading, alternating with loosely arranged foci (Antoni B). Divergent differentiation, either epithelial or mesenchymal, is an infrequent finding in benign nerve sheath tumors. The occurrence of signet-ring cells in benign nerve sheath tumors is exceedingly rare (16). To date, only 7 cases of lipoblastic nerve sheath tumors displaying signet-ring cells have been documented in the scientific literature (**Table 1**). In 2006, Plaza et al. first reported 5 cases of benign nerve sheath neoplasms that displayed prominent signet-ring cells with lipoblast-like features (1). Vecchio et al. described one additional case of neurofibroma exhibiting lipoblast-like signet-ring cells in 2010 (17). None of the six documented lipoblastic nerve sheath tumors showed cellular atypia. In 2012, However, Fedda et al. reported the first instance of lipoblastic nerve sheath tumor with degenerative atypia and multivacuolated lipoblasts (18). it has been reported that lipoblasts and atypical lipomatous-like regions appear more frequently in malignant solitary fibrous tumor (SFT) and may prompt a careful search for morphologic evidence of malignancy in fat-forming SFT (19). Including our case, all of the six patients for whom follow-up was available were alive with no evidence of recurrence over a period of 24 to 120 months (**Table 1**). Therefore, the presence of lipoblast-like cells may not indicate an adverse prognosis for patients with lipoblastic nerve sheath tumors. However, due to the limited number of cases reported, more case of this rare tumor with follow up need to be documented for further assessment of prognosis. Lipoblastic nerve sheath tumors have been documented to arise in various anatomic locations, including the thigh, groin, shoulder, and retroperitoneum. However, to the best of our knowledge, this neoplasm has yet to be reported as a lymph node primary, displaying with lipoblast-like cells and cellular atypia.

**Table 1:** Clinicopathologic features of reported peripheral nerve tumors displaying lipoblast-like cells.

Year	Author	Location	Size	Histology	IHC positive	IHC negative	Follow-up
2006	Plaza	Left groin	8×5×3.5 cm	Schwannoma	S100, Vimentin	CD34, SMA, Desmin, EMA, CD56, CEA, CK, Bcl-2	A&W at 29 months
2006	Plaza	Right thigh	6×5.5×4.5 cm	Schwannoma	S100, Vimentin	CD34, SMA, Desmin, EMA, CD56, CEA, CK, Bcl-2	A&W at 28 months
2006	Plaza	Right thigh	10×5×2.5 cm	Neurofibroma	S100, Vimentin	CD34, SMA, Desmin, EMA, CD56, CEA, CK, Bcl-2	A&W at 30 months
2006	Plaza	Retroperitoneum	12×8×6.5 cm	Schwannoma with degenerative changes	S100, Vimentin	CD34, SMA, Desmin, EMA, CD56, CEA, CK, Bcl-2	A&W at 116 months
2006	Plaza	Right shoulder	6×3×2.5 cm	Schwannoma with degenerative changes	S100, Vimentin	CD34, SMA, Desmin, EMA, CD56, CEA, CK, Bcl-2	NA
2010	Vecchio	Cervical region	6 cm	Neurofibroma	S100	CD34, panCK, Desmin, EMA, SMA	A&W at 24 months
2012	Fedda	Right thigh	2 cm	Neurofibroma with cellular atypia	S100, CD34, P16	SMA, MDM2,	NA
2023	Chen	Right inguinal lymph node	5.4×2.2×1.9 cm	Schwannoma with cellular atypia	S100, Vimentin, SOX10, CD56, NSE, MelanA	SMA, CK, EMA, Desmin, SMA, MDM2, HMB45, NF, p53	A&W at 120 months

A&W, alive and well, no evidence of recurrence; NA, not available.

This case report highlights three confounding features that can potentially obscure the diagnosis of benign lipoblastic nerve sheath tumors. Firstly, the tumor was located solely in the inguinal lymph node without any other lesions detected. Secondly, both monovacuolated and multivacuolated lipoblasts were present within the benign nerve sheath tumor. Lastly, the focal atypical cytology that sometimes manifested in nerve sheath tumors, can also contribute to the overdiagnosis of malignancy.

In terms of differential diagnoses, the main considerations are malignant peripheral nerve sheath tumor (MPNST), Signet-ring cell melanoma, atypical lipomatous/well-differentiated liposarcomas (ALT/WDLPS) and intranodal palisading myofibroblastoma (IPM). Typically, MPNSTs exhibit areas of geographical necrosis and marked mitotic activity. The absence of H3K27me3 expression is a valuable diagnostic marker for high-grade MPNSTs. Signet-ring cell melanoma is a seldom-observed histological subtype of malignant melanoma that manifests as a solid, nest-like growth pattern composed of round or spindle-shaped cells (20). These cells are frequently characterized by centrally-located nucleoli that coexist with numerous small and intermediate cells that demonstrate the hallmark signet-ring morphology. Metastatic melanoma presenting as a soft tissue mass is a seldom encountered scenario. The case under our scrutiny can be distinguished from melanoma due to the absence of necrosis and the characteristic nested arrangement of melanoma cells. While the tumor in question displays partial positivity for Melan-A, HMB45, a specific melanocytic marker, tested negative in the tumor. In addition, the neural-specific immunolabelling, such as S-100, SOX10, NSE, CD56, was not only strong but also diffusely positive.

ALT/WDLPS is characterized by specific genetic alterations, the amplification of the chromosomal region 12q13-15 harboring MDM2, CDK4, HMGIC and others genes, often exhibited the presence of ring and giant marker chromosomes (21). In clinical practice, the diagnosis of ALT/WDLPS often relies on the detection of the amplification and overexpression of MDM2 and CDK4 (22–25). However, several studies have reported that MDM2 or CDK4 can also be present in fibrosarcomas, leiomyosarcomas (LMS), osteosarcomas, rhabdomyosarcomas (RMS), and chondrosarcomas, as well as MPNSTs (26–28). Oliveira et al. conducted an immunohistochemical (IHC) study involving 129 soft tissue tumors. Their findings indicated that the overexpression of MDM2 and CDK4, can serve as a valuable tool for the diagnosis of WDLPS/DDLPS (29). In this case, the immunohistochemical analysis showed negative expression of MDM2 and scattered positive expression of CDK4, and the FISH analysis did not detect the amplification of MDM2 and CDK4 genes. Therefore, the diagnosis of WDLPS can be ruled out. Given that spindle-shaped cells within the tumor in our reported case can form a palisaded arrangement, closely resembling the morphology of IPM (30). However, the immunohistochemical analysis demonstrated a diffuse and strong positive for S-100, SOX10, H3K27me3, negative for SMA in tumor cells. Adversely, immunostaining usually positive for SMA and negative for S-100 in IPM. Moreover, the presence of Antoni type A and B areas and the absence of “amianthoid fibers” structures enable us to exclude the diagnosis of IPM. The diagnostic challenge posed by tumors with lipoblastic-like signet-ring cells, such as the one presented here, underscores the importance of immunohistochemical and histochemical analyses. Heightened awareness of the possibility of this exceedingly uncommon tumor can aid in distinguishing primary lipoblastic nerve sheath tumors in inguinal lymph nodes from other benign intranodal schwannomas, IPM and metastatic tumors.

Whole-exome sequencing analysis of this neoplasm identified six nonsynonymous SNV genes and four nonsense mutation genes. Interestingly, when the 10 mutated genes in lipoblastic nerve sheath tumors were subjected to KEGG analysis, the results revealed that these genes were mainly enriched in two pathways: EGFR tyrosine kinase inhibitor resistance and the MAPK signaling pathway. And we observed an overlap in mutated genes between lipoblastic nerve sheath tumors and MPNST, with both showing mutations in the *OTOA* and *NF1* genes. As we known, *NF1* is a tumor suppressor gene, which function of loss could be caused by nonsense mutation, and EGFR or MAPK signaling involved in schwann cell tumors and MPNSTs (31, 32),However, what roles they played in the oncogenesis and development of lipoblastic nerve sheath tumor are still unknown. More cases need to be accumulated for further investigation the underlying mechanisms of this rare neoplasm.

In summary, our case report may potentially represent the first documented instance of a lipoblastic nerve sheath tumor as a lymph node primary. Though the presence of lipoblastic-like signet-ring cells make the diagnosis challenging, immunohistochemical and FISH analyses, as well as whole-exome sequencing be utilized to aid in the identification of this rare tumor. The recognition of this unusual presentation can assist the practicing pathologist avert an erroneous diagnosis of lipoblastic nerve sheath tumor.

## Data availability statement

The original contributions presented in the study are included in the article/**Supplementary Material**. Further inquiries can be directed to the corresponding authors.

## Ethics statement

The studies involving humans were approved by the Ethics Committee of the First Affiliated Hospital of Shihezi University School of Medicine (Approval number: 2019-021-01). The studies were conducted in accordance with the local legislation and institutional requirements. The human samples used in this study were acquired from primarily isolated as part of previous study for which ethical approval was obtained. Written informed consent was obtained from the participant for the publication of this case report.

## Author contributions

CC: Data curation, Writing – original draft, Writing – review & editing, Formal Analysis. JC: Writing – review & editing. LS: Data curation, Writing – review & editing. WW: Writing – original draft. DG: Data curation, Writing – review & editing. QS: Software, Writing – review & editing. YZ: Investigation, Writing – review & editing. YC: Supervision, Visualization, Writing – review & editing. CL: Conceptualization, Funding acquisition, Methodology, Writing – review & editing. FL: Conceptualization, Data curation, Funding acquisition, Writing – original draft, Writing – review & editing.
