# Supplementary material for: Primary lipoblastic nerve sheath tumor in an inguinal lymph node mimicking metastatic tumor: a case report and literature review
Source: Front Oncol. 2023 Oct 30;13:1258769. doi: 10.3389/fonc.2023.1258769 (PMC10642330; doi:10.3389/fonc.2023.1258769)
Supplement: Supplementary file 1 [file DataSheet_1.docx]

Supplementary Materials

# Supplementary Figure 1.

**
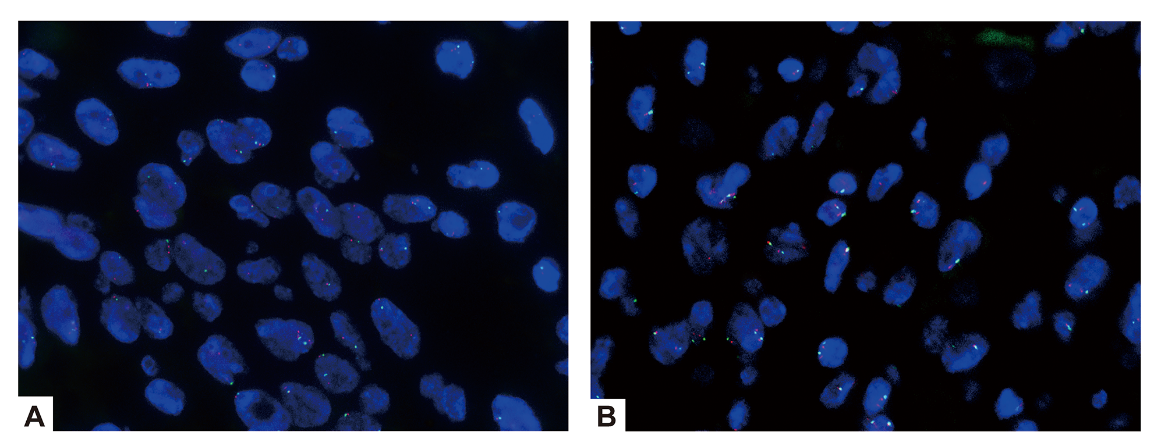
**

**Supplementary Fig 1. FISH for amplification of MDM2 and CDK4 in the lipoblastic nerve sheath tumor.**

In the FISH analysis, this case does not exhibit amplification of either MDM2 (A: red signal, MDM2; green signal, CEP12, original magnification×1000) or CDK4 (B: red signal, CDK4; green signal, CEP12, original magnification×1000).

# Supplementary Figure 2.


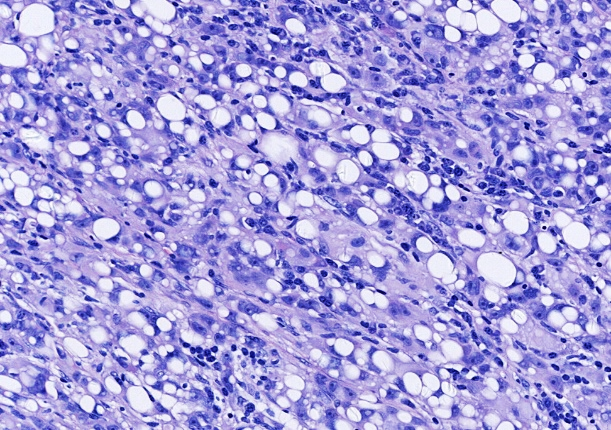

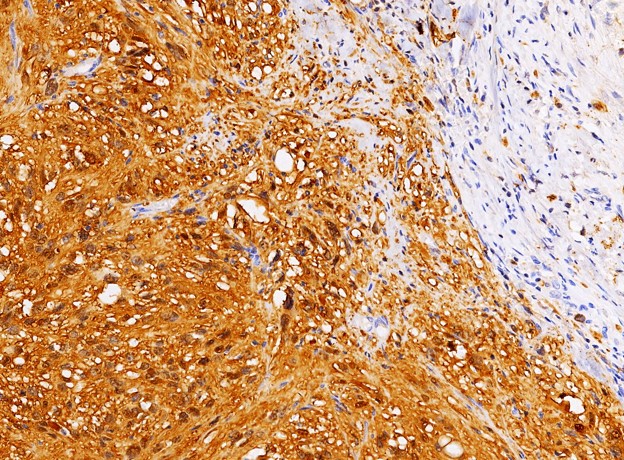


**B**

**A**

**Supplementary Fig 2. adipocytic differentiation in the tumor.** A. adipose cells (HE×200). B. S-100 highlighted both lipoblasts and spindle cells (HE×200).

# Supplementary Figure 3.


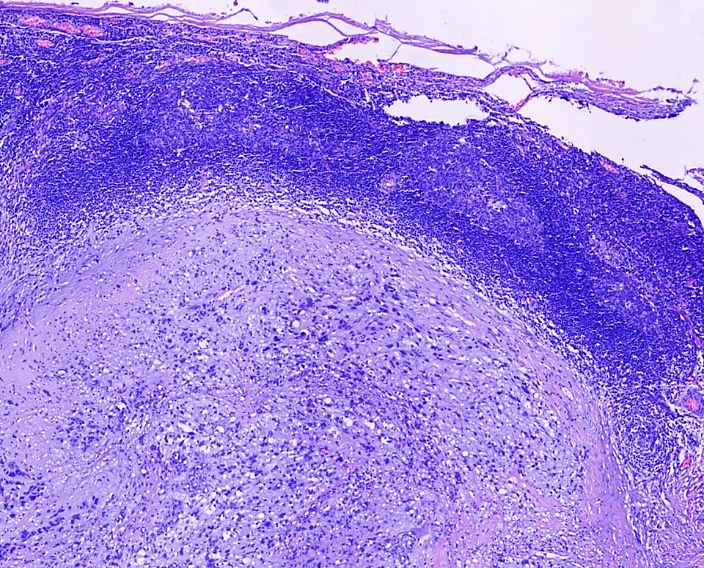

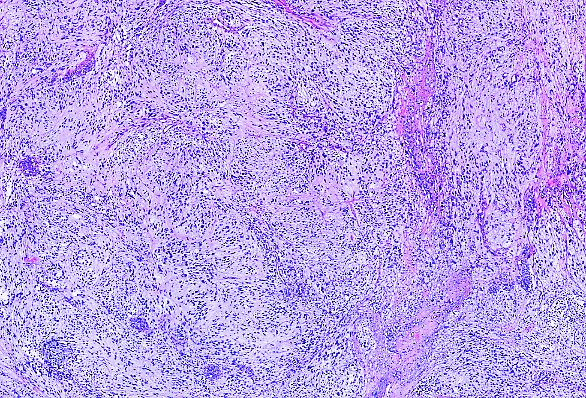

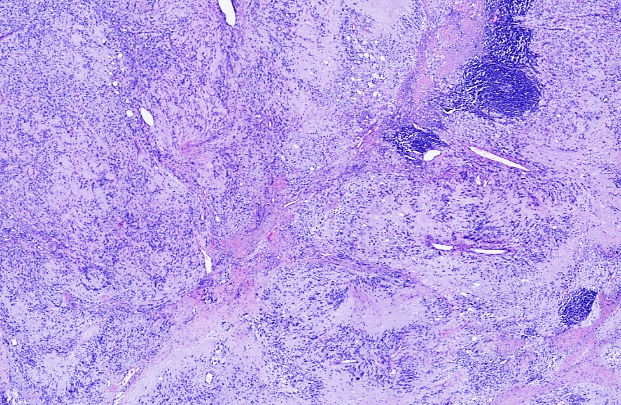

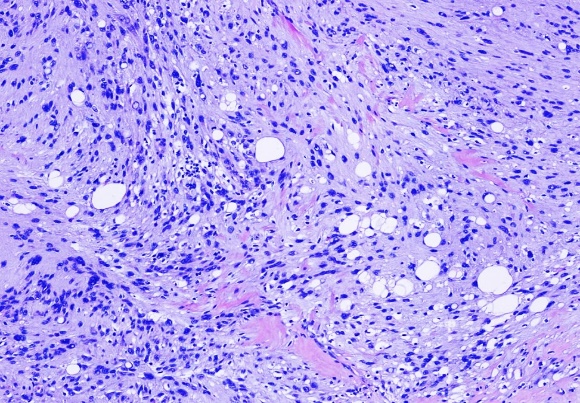


**A**

**D**

**C**

**B**

**Supplementary Fig 3. Morphological features of schwannoma.**

A.The nodular tumor with fibrous pseudocapsule (HE×80). B.C. Typical area of schwannoma shows a variably admixture of zones of high and low cellularity at low magnification, hyalinized thick-walled vessels are seen (HE×40). D. Cytologic feature of tumor cells (HE×100).

# Supplementary Figure 4.

**
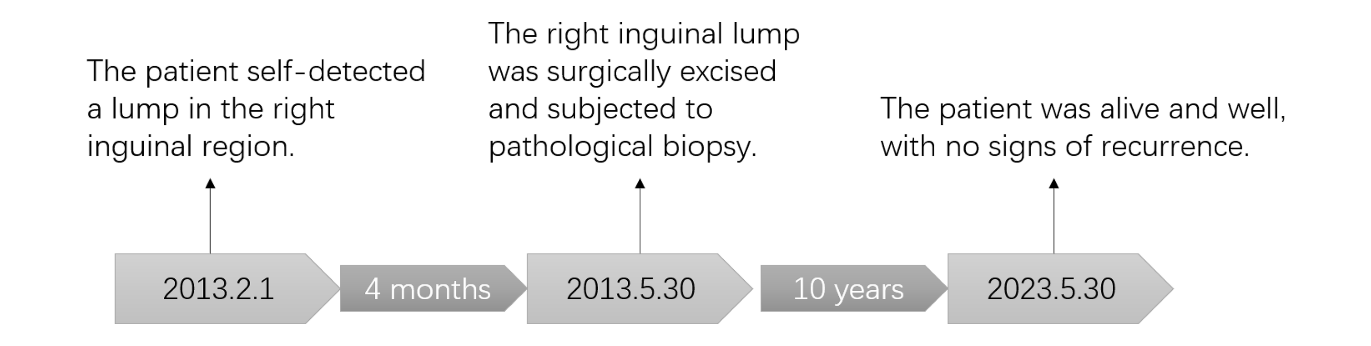
**

**Supplementary Fig 4. The disease progression timeline.**
